# Burkitt Lymphoma With Aberrant Expression of Cytoplasmic Terminal Deoxynucleotidyl Transferase: A Case Report

**DOI:** 10.7759/cureus.53972

**Published:** 2024-02-10

**Authors:** Mark T Cunningham, Carmen Winters, Daniel Farrell

**Affiliations:** 1 Pathology and Laboratory Medicine, University of Kansas Medical Center, Kansas City, USA; 2 Pathology, Cascade Pathology Services, Portland, USA

**Keywords:** flow cytometry, immunophenotype, immunoperoxidase, immunohistochemistry, terminal deoxynucleotidyl transferase, burkitt lymphoma

## Abstract

This study describes a rare case of Burkitt lymphoma with aberrant expression of cytoplasmic terminal deoxynucleotidyl transferase (TdT). Flow cytometry demonstrated a predominantly mature B cell immunophenotype as expected for Burkitt lymphoma, however, the immature marker TdT was also expressed. Immunohistochemistry showed that TdT was localized to the cytoplasm, with absent nuclear localization. The patient received standard intensive chemotherapy for Burkitt lymphoma and has remained in remission for nine years. Pathologists should be aware of this unusual phenomenon of aberrant cytoplasmic TdT expression to avoid confusing Burkitt lymphoma with B cell lymphoblastic leukemia/lymphoma.

## Introduction

Burkitt lymphoma is an aggressive mature B cell neoplasm accounting for 1-2% of total non-Hodgkin lymphoma diagnoses among adults in the United States [1-2]. There are three distinct epidemiological variants: endemic, sporadic, and immunodeficiency-associated [3]. The pathogenesis involves the translocation of the MYC protooncogene on chromosome 8 [4]. MYC gene fusion usually occurs with the IGH gene on chromosome 14, and less frequently with either the IGK gene or IGL gene on chromosomes 2 and 22, respectively.

The pathological diagnosis of Burkitt lymphoma requires a multiparametric approach, including morphology, flow cytometry, immunohistochemistry, cytogenetics, and fluorescence in situ hybridization [3]. Morphologically, the lymphoma cells show French-American-British (FAB) L3 morphology: monomorphic medium-sized nuclei, non-cleaved nuclear contours, dispersed chromatin, multiple medium-sized nucleoli, moderate deeply basophilic cytoplasm, and multiple lipid-filled vacuoles that have a “paper punch” appearance and overlie the nucleus. Histological sections show a diffuse infiltrate of lymphoma cells, scattered tingible body macrophages (“starry sky” pattern), increased mitotic figures, and increased apoptosis.

Flow cytometric and immunohistochemical analysis of Burkitt lymphoma reveals a mature B cell immunophenotype [3]. The lymphoma cells are positive for CD10, CD19, CD20, CD22, bright CD45, CD79a, BCL6, Ki67 (> 95%), MYC, PAX5, and monoclonal surface immunoglobulin. They are usually negative for BCL2 and typically do not express markers of B cell immaturity such as CD34, dim CD45, and nuclear TdT. 

TdT is a member of the X family of DNA polymerases and functions by adding nucleotides to the 3’ end of single-stranded DNA [5]. This enzyme is normally expressed by progenitor B cells in the bone marrow and progenitor T cells in the bone marrow and thymus. TdT plays an important role in V(D)J recombination, which allows the immense diversity of immunoglobulins and T cell receptors of the normal immune system. B cell lymphoblastic leukemia/lymphoma and T cell lymphoblastic leukemia/lymphoma are two neoplasms that are derived from progenitor B cells and progenitor T cells, respectively. These neoplasms express TdT in 95% of cases, and TdT expression appears to be exclusively localized to the nucleus [6-8]. 

To our knowledge, the expression of cytoplasmic TdT has not been reported in Burkitt lymphoma. We report a case of Burkitt lymphoma that aberrantly expresses cytoplasmic TdT. This phenomenon was observed by both flow cytometry and immunohistochemistry. This study describes the clinical, morphologic, immunophenotypic, and cytogenetic features of this unusual manifestation of Burkitt lymphoma.

## Case presentation

A 21-year-old female presented with a one-month history of fatigue, myalgia, night sweats, and a 30-pound unintentional weight loss. The physical exam was unremarkable. Computed tomography (CT) of the chest showed an 8.4 cm mediastinal mass, bilateral hilar lymphadenopathy, and bilateral axillary lymphadenopathy. Laboratory studies are summarized in Table 1.

**Table 1:** Pertinent laboratory results EBV: Epstein Barr virus

Laboratory Parameter	Result	Reference Range
Hemoglobin	6.9 g/dL	12.0-15.0 g/dL
Platelets	107,000 /uL	150,000-400,000 /uL
White blood cell count	5,200 /uL	4,500-11,000 /uL
Neutrophil count	3,120 /uL	1,800-7,000 /uL
Lymphocyte count	1,664 /uL	1,000-4,800 /uL
Monocyte count	104 /uL	0-800 /uL
Eosinophil count	52 /uL	0-450 /uL
Peripheral blood lymphoma cells	5%	0%
Lactate dehydrogenase	2,130 IU/L	100-190 IU/L
Alanine aminotransferase	28 IU/L	7-56 IU/L
Aspartate aminotransferase	35 IU/L	7-40 IU/L
Alkaline phosphatase	77 IU/L	25-110 IU/L
Creatinine	0.59 mg/dL	0.40-1.00 mg/dL
Uric acid	5.0 mg/dL	2.0-6.0 mg/dL
Phosphorus	3.7 mg/dL	2.0-4.0 mg/dL
Potassium	2.8 mmol/L	3.5-5.1 mmol/L
Calcium	8.6 mg/dL	9.0-11.0 mg/dL
Corrected calcium	9.7 mg/dL	9.0-11.0 mg/dL
Albumin	2.6 g/dL	3.5-5.0 g/dL
Blood EBV quantitative DNA	Negative	Negative
Cerebrospinal fluid cytology	Negative	Negative

**Figure 1:**
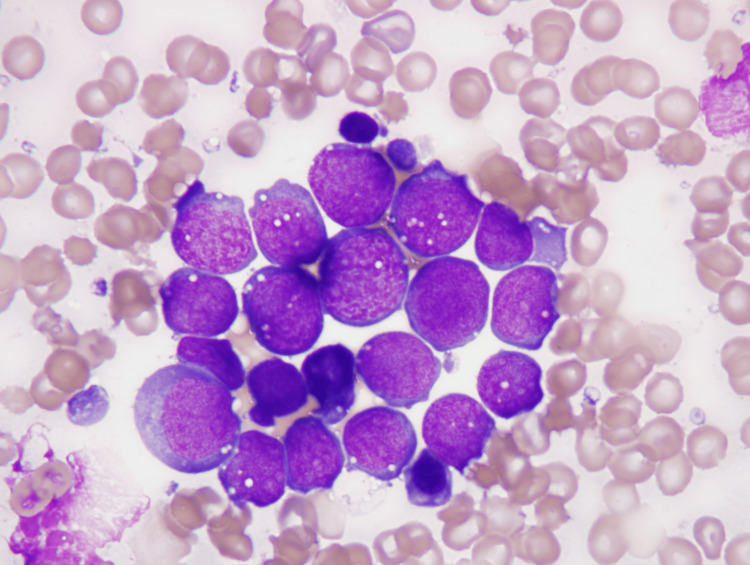
Bone marrow touch prep showing lymphoma cells with French-American-British (FAB) L3 morphology (Wright stain, 1000x)

Bone marrow aspiration resulted in a dry tap. Bone marrow biopsy touch prep showed 90% lymphoma cells with the following morphological features: monomorphic, medium-sized nuclei, non-cleaved nuclear contours, dispersed chromatin, several small to medium-sized nucleoli, deeply basophilic cytoplasm, and prominent “paper punch” vacuoles overlying the nucleus (Figure 1).

**Figure 2:**
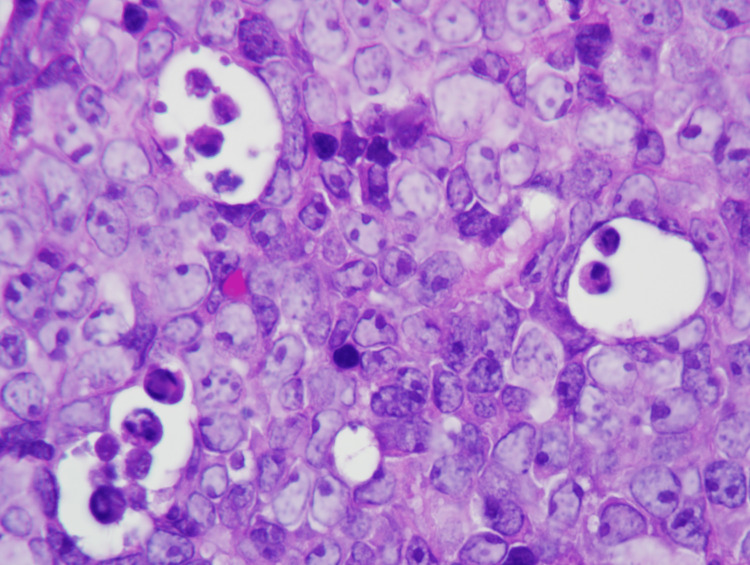
Bone marrow core biopsy showing a diffuse cellular infiltrate of lymphoma cells with a “starry sky” pattern (H&E stain, 500x)

Bone marrow biopsy revealed a hypercellular marrow (90%), diffuse lymphoma infiltrate, and increased tingible body macrophages with a “starry sky” pattern (Figure 2).

**Figure 3:**
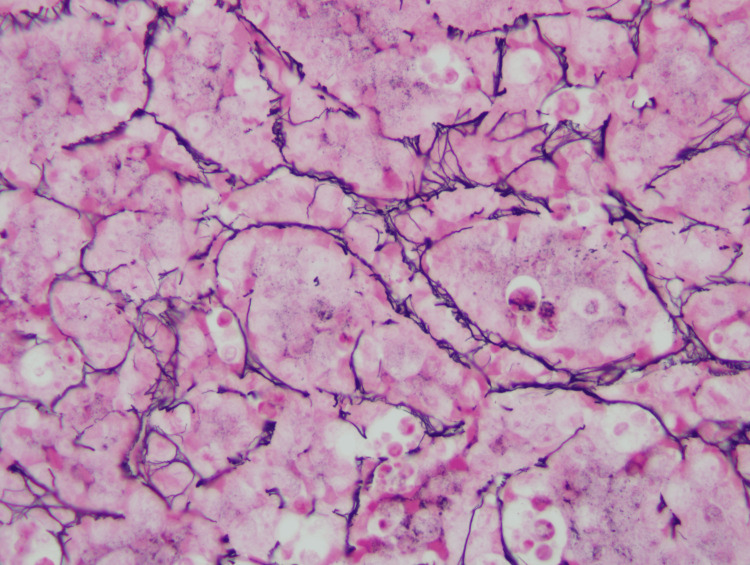
Bone marrow core biopsy showing moderate diffuse reticulin fibrosis (Reticulin stain, 500x)

Reticulin stain on the bone marrow biopsy showed moderate diffuse reticulin fibrosis (Figure 3).

Flow cytometry revealed a mature B cell immunophenotype. The lymphoma cells (58% of total events) were positive for CD10, CD19, CD20, bright CD45, FMC7, and monoclonal surface immunoglobulin kappa light chain. The lymphoma cells were negative for CD5, CD23, and CD34. The single unusual finding was positivity for intracellular TdT, which is a marker of B cell immaturity. The anti-TdT antibody used for flow cytometry was a mouse anti-human monoclonal antibody (manufacturer: SuperTechs Inc, Gaithersburg, Maryland; product name: TdT-6; clone name HT-6; fluorophore: FITC; dilution 1:10), and cells were permeabilized during antibody labeling using the Invitrogen Fix and Perm cell permeabilization kit. 

**Figure 4:**
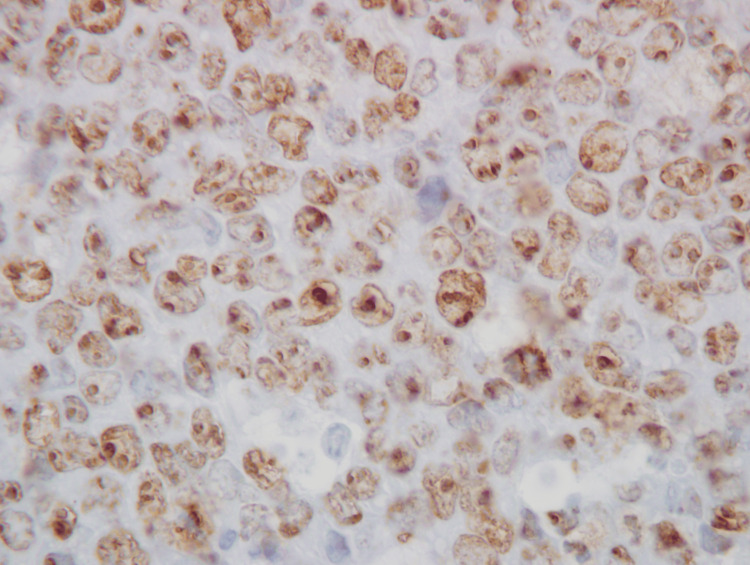
Bone marrow core biopsy showing a Ki67 proliferation index of > 95% (Ki67 immunoperoxidase stain, 500x)

Immunohistochemistry was performed on the bone marrow core biopsy for BCL2, BCL6, CMYC, Ki67, and TdT. Chromogenic in situ hybridization was performed on the core biopsy for EBV. Staining was performed on formalin-fixed paraffin-embedded tissue. The lymphoma cells were positive for BCL6, CMYC, Ki67 (> 95%), and TdT. The lymphoma cells were negative for BCL2 and EBV. The Ki67 stain is shown in Figure 4.

**Figure 5:**
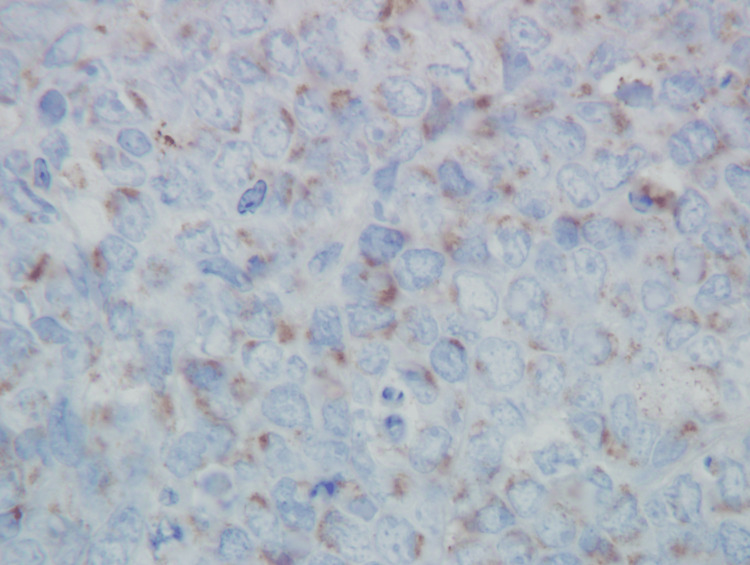
Bone marrow core biopsy showing TdT expression in 95% of lymphoma cells in a cytoplasmic granular pattern (TdT immunoperoxidase stain, 500x)

The TdT stain is shown in Figure 5.

TdT was expressed in 95% of 100 cells examined. The pattern of expression was cytoplasmic, with most cells containing a single large cluster of granules, some cells containing two large clusters of granules, and infrequent cells containing multiple scattered small granules. There was no evidence of a nuclear expression pattern. The anti-TdT antibody used for immunohistochemistry was a rabbit anti-human monoclonal IgG antibody (manufacturer: Agilent, Santa Clara, California; product name: TdT; clone name: EP266; prediluted; antigen retrieval: high pH target retrieval solution used as directed). 

Conventional cytogenetics and fluorescence in situ hybridization on the bone marrow are shown in Table 2.

**Table 2:** Bone marrow cytogenetic and fluorescence in situ hybridization analysis

Laboratory Parameter	Result	Reference Range
Conventional karyotype	47,X,add(X)(p11.2),del(6)(q13),der(8)t(8;14)(q24.1;q32),der(14)(pter-> q32::8q24.1::22q11.2-> 22qter),+10,der(22)(pter-> q11.2::8q24.1-> 8qter)(15 metaphases)/46,XX(5 metaphases)	46,XX (20 metaphases)
Fluorescence in situ hybridization for MYC (8q24) rearrangement	42.5 % interphase nuclei positive	0 % interphase nuclei positive
Fluorescence in situ hybridization for BCR (22q11.2) and ABL1 (9q34) rearrangement	0 % interphase nuclei positive	0 % interphase nuclei positive

The patient was diagnosed with Burkitt lymphoma and was treated with eight cycles of hyper-CVAD (cyclophosphamide, vincristine, doxorubicin, dexamethasone) alternating with methotrexate and cytarabine. The patient went into complete remission based on a repeat CT scan and repeat bone marrow biopsy and has remained in complete remission for nine years at the most recent follow-up visit.

## Discussion

To our knowledge, this is the first reported case of Burkitt lymphoma with cytoplasmic TdT expression. This phenomenon was observed by both flow cytometry and immunohistochemistry. The anti-TdT monoclonal antibodies used by flow cytometry and immunohistochemistry were different clones, which validates the TdT specificity of antibody reactivity by the two different methods. TdT expression was localized to the cytoplasm by immunohistochemistry, and there was absent nuclear expression. The cytoplasmic reactivity pattern was predominantly “large granular,” which manifested as one or two large clusters of granules.

A limited number of normal cell types have been shown to express cytoplasmic TdT. Gregoire et al. reported that immature T cells in the thymus gland have predominantly cytoplasmic TdT expression [9]. Park et al. reported that 1-2% of TdT-positive precursor B cells in the mouse bone marrow are in metaphase and have cytoplasmic TdT expression [10].

Some neoplasms also rarely express cytoplasmic TdT. Henwood reported a combination of “muddy” homogenous cytoplasmic TdT expression and nuclear TdT expression in five cases of B cell lymphoblastic lymphoma and three cases of T cell lymphoblastic lymphoma [11]. This staining pattern was specifically seen on tissue that had been frozen for cryotomy and subsequently, formalin-fixed and paraffin-embedded. In contrast, nuclear TdT expression was predominantly seen on the same tissues that were immediately fixed in formalin. This phenomenon was termed “cryotomy-induced antigen diffusion”, and it did not occur in cases of Burkitt lymphoma or diffuse large B cell lymphoma. Cibull et al. reported two cases of chronic myeloid leukemia in blast crisis with a combination of cytoplasmic TdT expression and nuclear TdT expression [12].

B cell neoplasms with Burkitt-like morphology, MYC rearrangement, and immature B cell immunophenotype have been previously reported [13-19]. These cases had variable markers of immaturity including positivity for CD34 [17], positivity for dim CD45 [15-16], positivity for TdT [13-16,18-19], negativity for CD20 [13-15,17-19], and negativity for surface immunoglobulin [13-14, 17-19]. Each of these cases had two or more markers of immaturity in those studies where TdT and surface immunoglobulin were included in the immunophenotype analysis. In addition, the TdT expression pattern was always nuclear by immunohistochemistry [13,16,19]. Our case was unique in that TdT was the only marker of immaturity present and the expression pattern was cytoplasmic.

One limitation of our study was that we were unable to completely exclude the possible diagnosis of high-grade B cell lymphoma with MYC rearrangement combined with BCL2 rearrangement and/or BCL6 rearrangement. There was no formalin-fixed paraffin-embedded tissue on the bone marrow aspirate due to the dry tap, which prevented retrospective fluorescence in situ hybridization analysis for BCL2 and BCL6. We think this diagnosis was unlikely since BCL2 and BCL6 rearrangements were not identified by conventional cytogenetics, and the patient responded well to standard therapy for Burkitt lymphoma.

Pathologists should be aware of this unusual phenomenon of cytoplasmic TdT expression to avoid confusing the diagnosis of Burkitt lymphoma with B cell lymphoblastic leukemia/lymphoma. In our case, the diagnosis of Burkitt lymphoma was strongly favored due to FAB L3 morphology, high Ki67 proliferation index (> 95%), t(8;14) MYC rearrangement, and mature B cell immunophenotype in all respects other than cytoplasmic TdT expression. Specifically, the lymphoma cells in our case were positive for the mature B cell markers CD20, bright CD45, and surface immunoglobulin, and negative for the immature B cell markers CD34 and dim CD45. We recommend that if intracellular TdT is identified by flow cytometry in the diagnostic workup of a malignant lesion suspicious for Burkitt lymphoma, immunohistochemistry should be performed to verify the accuracy of the flow cytometry result and to determine the cellular localization of TdT.

## Conclusions

This case report demonstrated that Burkitt lymphoma can rarely express cytoplasmic TdT. We showed that immunohistochemistry was useful in characterizing this unusual cellular expression pattern. Aberrant cytoplasmic TdT expression in this case did not predict a worse prognosis. This patient had an excellent outcome following standard therapy for Burkitt lymphoma.
